# Clinical outcomes of esophageal squamous cell carcinoma in patients with cirrhosis

**DOI:** 10.1016/j.ctro.2024.100817

**Published:** 2024-07-07

**Authors:** Dae Gon Ryu, Mi Sook Yun, Hongqun Liu, Samuel S. Lee, Sangjune Laurence Lee

**Affiliations:** a Liver Unit, University of Calgary Cumming School of Medicine, Calgary, AB, Canada; b Department of Internal Medicine, Pusan National University School of Medicine and Research Institute for Convergence of Biomedical Science and Technology, Pusan National University Yangsan Hospital, Yangsan, Republic of Korea; c Devision of Biostatistics, Research Institute for Convergence of Biomedical Science and Technology, Pusan National University Yangsan Hospital, Yangsan, Republic of Korea; d Division of Radiation Oncology, University of Calgary, Tom Baker Cancer Centre, Calgary, AB, Canada

**Keywords:** Esophageal cancer, Squamous cell carcinoma, Cirrhosis

## Abstract

**Purpose:**

Alcohol consumption is a strong risk factor for both cirrhosis and esophageal squamous cell carcinoma (ESCC). Few studies have been conducted on the treatment of ESCC in patients with cirrhosis. This study aimed to analyze the clinical outcomes of ESCC in patients with cirrhosis.

**Materials and methods:**

Medical records of patients with esophageal cancer between January 2009 and December 2023 were retrospectively reviewed. A total of 479 patients with ESCC were included and divided into cirrhotic (n = 69) and non-cirrhotic (n = 410) groups. Clinical outcomes and survival according to treatment were compared between these groups.

**Results:**

The cirrhotic group was younger (median age 64 years vs. 69 years, *p* = 0.022) and had a higher proportion of male (97.1 % vs. 88.3 %, *p* = 0.042) than the non-cirrhotic group. Patients with cirrhosis were less likely to undergo surgery (31.9 % vs. 47.8 %, *p* = 0.015) and were more likely to receive no active cancer treatment (26.1 % vs. 13.7 %, *p* = 0.010). Overall survival was lower in the cirrhotic group (hazard ratio [HR], 1.41; 95 % confidence interval [CI], 1.01–1.99; *p* = 0.045), however, no difference was found between Child-Pugh class A patients and those in the non-cirrhotic group (HR, 1.04 [95 % CI, 0.69–1.56]; *p* = 0.864). Postoperative mortality was significantly higher in cirrhotic group (27.3 % vs. 8.7 %, *p* = 0.011). Upon performing concurrent chemoradiotherapy (CRT), the clinical complete response rate (84.2 % vs. 43.3 %, *p* = 0.004) was better in the cirrhotic group. CRT yielded better overall survival for patients with cancer in the resectable stages in the cirrhotic group compared to surgery (HR, 0.19 [95 % CI, 0.42–0.84]; *p* = 0.029].

**Conclusions:**

In patient with ESCC and cirrhosis, chemoradiotherapy may be a better treatment option than surgery.

## Highlights

• Alcohol consumption is a significant risk factor for both esophageal squamous cell carcinoma (ESCC) and cirrhosis.
• Overall, patients with ESCC and cirrhosis have a worse prognosis than patients with ESCC but without cirrhosis.
• The subgroup of patients with ESCC and Child–Pugh class A have similar outcomes to patients with ESCC but without cirrhosis.
• Patients with ESCC and cirrhosis have poorer outcomes and survival after surgery compared to after chemoradiotherapy.

## 1 Introduction

Esophageal cancer ranks as the eighth most common cancer and the sixth most common cancer-related death worldwide [1]. It carries a poor prognosis and predominantly affects the elderly, contributing to its increasing incidence globally alongside rising life expectancies [2]. The primary histological types of esophageal cancer are squamous cell carcinoma (SCC) and adenocarcinoma (AC). Despite increasing prevalence of AC, SCC remains the most common histological type globally [1]. SCC exhibits a strong association with alcohol and tobacco use and is notably sensitive to radiation therapy.

While surgery stands as a curative option for esophageal cancer, concurrent chemoradiotherapy (CRT) also proves effective, with neoadjuvant CRT considered the standard for locally advanced cases [3]. When surgery is performed after neoadjuvant CRT for SCC, the pathological complete response rate is more than 40 % [4], [5]. Hence, the guidelines recommend that in SCC cases, observation instead of surgery is possible when no evidence of disease remains after neoadjuvant CRT [3].

Cirrhosis and esophageal cancer, particularly SCC, share alcohol as a significant risk factor. It has been reported that the incidence of esophageal malignancy is approximately eight times higher in patients with cirrhosis [6]. However, few studies have investigated the management of esophageal cancer in patients with cirrhosis. Some surgical studies have concluded that esophagectomy in patients with cirrhosis is associated with high morbidity and mortality [7], [8]. Studies on treatments other than surgery for esophageal cancer in patients with cirrhosis, such as CRT or endoscopic resection, are limited to case reports or series [9], [10], [11]. This retrospective observational study aimed to identify clinical outcomes and survival of patients with ESCC and cirrhosis.

## 2 Material and methods

### 2.1 Patients

The medical records of patients treated between December 2008 and December 2023 at a single academic hospital were reviewed retrospectively. During the study period, 479 patients with histologically confirmed esophageal SCC (ESCC) who underwent follow-up were included. Esophageal cancer was staged according to the American Joint Committee on Cancer (AJCC) staging system, revision 8th edition [12]. This study was approved by the Ethics Committee of our center’s review board (institutional review board no. 55–2023-07). The ethics committee waived the requirement for informed consent since participants’ medical records were anonymized prior to analysis.

### 2.2 Cirrhosis

Patients with cirrhosis were defined as those who were clinically already diagnosed with cirrhosis and exhibited overt imaging findings of cirrhosis on ultrasound or computed tomography (CT). The cause of cirrhosis was classified based on medical history and serum markers of hepatitis, and its severity was classified using the Child’s–Pugh (C–P) score [13].

### 2.3 Surgery

Patients selected for surgery underwent Ivor Lewis esophagectomy with intrathoracic anastomosis and two- or three-field lymph nodes dissections in the absence of anatomical abnormalities. Patients with anatomical abnormalities such as gastrectomy underwent colonic or jejunal interposition. Pathological analysis evaluated completeness of resection (R0) and lymph node metastasis. Anastomotic leakage was assessed 1 week after surgery using endoscopy and esophagography, with postoperative mortality defined as mortality within 30 days after surgery or during postoperative hospitalization.

### 2.4 Chemoradiotherapy

CRT was categorized into definitive CRT, which was performed with curative intent, and neoadjuvant CRT prior to surgery. Depending on the patient's condition, some received definitive radiation therapy (RT) only, while others underwent palliative RT for symptom relief. RT schedules and doses are as follows: (1) Definitive CRT, 2 Gy x 25 fractions for a total of 50 Gy; (2) neoadjuvant CRT, 1.8 Gy x 25 fractions for a total of 45 Gy; and (3) Palliative RT, 3 Gy x 10 fractions for a total of 30 Gy. Chemotherapy regimens employed for most patients were cisplatin/5-fluorouracil (cisplatin 75 mg/m^2^ for 1 day and 5-fluouracil 750 mg/m^2^/d for 4 days at weeks 1 and 5) or paclitaxel/carboplatin (paclitaxel 50 mg/m^2^ and carboplatin area under the curve of 2 mg/ml/min on day 1 weekly over 5 weeks).

### 2.5 Endoscopic resection

Some superficial esophageal cancers without evidence of lymph node metastasis on preoperative examination and localized to the mucosa on endoscopic ultrasound underwent endoscopic resection. Most patients underwent endoscopic submucosal dissection (ESD), and most procedures were conducted under conscious sedation via intravenous midazolam without general anesthesia. If no complications occurred, the patient was discharged 2 days postoperatively. Complete resection was defined as a single-piece resection without fragmentation and tumor-free margins on histological examination. Curative resection was defined as the absence of submucosal invasion, lymphovascular involvement, or poorly differentiated features in the resected specimen.

### 2.6 Follow-up after treatment

After curative treatment (surgery, definite CRT, or endoscopic resection), endoscopy and chest/abdominal CT were conducted were performed 2–3 months later, every 3 months for the first year, and every 6 months thereafter. Positron emission tomography (PET) was not conducted after surgery or endoscopic resection, whereas it was conducted 3–6 months after definitive CRT. Typically, surgery was performed 2 months after neoadjuvant CRT; however, some patients were observed without surgery according to preoperative examination results and the patient’s condition through a multidisciplinary approach. Clinically complete response (cCR) after CRT was defined as no evidence of cancer in examinations 2–3 months after completing CRT, including endoscopy, endoscopic biopsy, chest/abdominal CT, and PET. For ambiguous examination results due to esophagitis or ulcers following CRT, examinations were performed again after 2–3 months.

### 2.7 Statistical analysis

Student’s t–test, and chi-square test or Fisher’s exact test were utilized for continuous and categorical variables for in-group comparisons, respectively. The Kaplan–Meier method was utilized for estimating survival, and log-rank test was employed for determining significant differences between each group. A Cox regression model was utilized for estimating hazard ratio (HR) associated with survival. Statistical significance was set at *p* < 0.05. The Statistical Package for the Social Sciences (SPSS) version 27.0 (IBM Corp., Armonk, NY, USA) was utilized for statistical analyses.

## 3 Results

### 3.1 Characteristics of the patients

During the study period, 479 patients with histologically confirmed ESCC, were identified and divided into cirrhotic group and non-cirrhotic groups. There were 69 and 410 patients in the cirrhotic and non-cirrhotic groups, respectively. The cirrhotic group was younger at the time of diagnosis (median, 64 [range, 45–87] years vs. 69 [range, 41–95] years; *p* = 0.022), and the proportion of men was higher (97.1 % vs. 88.3 %, *p* = 0.042) compared to the non-cirrhotic group. Fifty-four patients (78.3 %) in the cirrhotic group had C–P class A disease. No differences were revealed in the location of the SCC in the esophagus or cancer stage between these groups. Patients in the cirrhotic group were less likely to undergo surgery (31.9 % vs. 47.8 %, *P* = 0.015), and were more likely to forgo active cancer treatment (26.1 % vs. 13.7 %, *P* = 0.010). The baseline characteristics of the two groups are summarized in Table 1. Alcohol consumption (n = 47, 68.1 %) was the most common etiology of cirrhosis, followed by hepatitis B virus (n = 8, 11.6 %), and hepatitis C virus (n = 6, 8.7 %). Esophageal varices were observed in 30 patients with cirrhosis. Four patients developed esophageal cancer after liver transplantation.

**Table 1:** Baseline characteristics of patients.

	**Cirrhosis** **(n = 69)**	**Non-Cirrhosis** **(n = 410)**	***p* value**
**Age, median (range) years**	64 (45 ∼ 87)	69 (41 ∼ 95)	*0.022*
**Male, n (%)**	67 (97.1)	362 (88.3)	*0.042*
**Child**–**Pugh Classification**			
*A*	54 (78.3)	non-applicable	
*B*	7 (10.1)	non-applicable	
*C*	8 (11.6)	non-applicable	
**Location, n (%)**			
*Cervical*	5 (7.2)	24 (5.9)	*0.654*
*Upper thoracic*	10 (14.5)	62 (15.1)	*0.892*
*Mid thoracic*	35 (50.7)	176 (42.9)	*0.229*
*Lower thoracic*	19 (27.5)	148 (36.1)	*0.169*
cStage*, n (%)			
*I*	18 (26.1)	105 (25.6)	*0.933*
*II*	15 (21.7)	77 (18.8)	*0.933*
*III*	23 (33.3)	138 (33.7)	*0.958*
*IV*	13 (18.8)	90 (22.0)	*0.561*
**Initially metastatic cancer, n (%)**	10 (14.5)	67 (16.3)	*0.699*
**Main treatment, n (%)**			
*Operation*	22 (31.9)	196 (47.8)	*0.015*
*Chemoradiotherapy or radiotherapy*	19 (27.5)	90 (22.0)	*0.307*
*Endoscopic resection*	8 (11.6)	46 (11.2)	*0.928*
*Chemotherapy*	2 (2.9)	22 (5.4)	*0.393*
*None*	18 (26.1)	56 (13.7)	*0.010*

*Clinical tumor–node–metastasis (cTNM) according to American Joint Committee on Cancer (AJCC) TNM version 8.

### 3.2 Surgery

A total of 218 patients underwent surgery, with 22 in the cirrhotic group and 196 in the non-cirrhotic group. All patients with cirrhosis who underwent surgery were C–P class A except two (90.1 %). No differences were found in age or pathological stage postoperatively between these groups. In patients with cirrhosis, tumor location was more frequent in the mid-thoracic esophagus (77.3 %), and all patients underwent an esophagogastric anastomosis. Neoadjuvant CRT was performed in five patients (22.7 %) and 29 patients (14.8 %) in the cirrhotic and non-cirrhotic groups, respectively. Despite a higher pathologic complete response (pCR) in the cirrhotic group compared to the non-cirrhotic group, the difference was not statistically significant due to limited number of patients (60.0 % vs. 37.9 %, *p* = 0.364). Similarly, R0 resection rate was higher in the cirrhotic group; however, this difference was not statistically significant (95.5 % vs. 87.8 %, *p* = 0.304). No significant difference was revealed in anastomotic leakage between the two groups (9.1 % vs. 9.7 %, *p* = 0.928); however, postoperative mortality was significantly higher in the cirrhotic group (27.3 % vs. 8.7 %, *p* = 0.011). A comparison between the two groups of patients who underwent surgery is summarized in Table 2.

**Table 2:** Comparison of two groups that underwent surgery.

	**Cirrhosis** **(n = 22)**	**Non-Cirrhosis** **(n = 196)**	***p* value**
**Age, median (range) years**	61 (51 ∼ 79)	65 (48 ∼ 90)	*0.285*
**Male, n (%)**	20 (90.1)	180 (91.8)	*0.881*
**Child**–**Pugh Classification, n (%)**			
*A*	20 (90.1)	non-applicable	
*B*	2 (9.1)	non-applicable	
*C*	0 (0)	non-applicable	
**Location, n (%)**			
*Cervical*	0 (0)	2 (1.0)	*0.727*
*Upper thoracic*	1 (4.5)	30 (15.3)	*0.201*
*Mid thoracic*	17 (77.3)	86 (43.9)	*0.005*
*Lower thoracic*	4 (18.2)	78 (39.8)	*0.057*
**Type of reconstruction**			
*Esophago-gastric anastomosis*	22 (100)	183 (93.4)	*0.411*
*Esophago-colonic anastomosis*	0 (0)	10 (5.1)	*0.526*
*Esophago-jejunal anastomosis*	0 (0)	3 (1.5)	*0.893*
**Neoadjuvant chemoradiotherapy**			
*Yes, n (%)*	5 (22.7)	29 (14.8)	*0.336*
Pathologic complete response*, n (%)	3/5 (60.0)	11/29 (37.9)	*0.364*
**R0 resection, n (%)**	21 (95.5)	172 (87.8)	*0.304*
**pStage^+^, n (%)**			
*0*	3 (13.6)	11 (5.6)	*0.160*
*I*	6 (27.3)	55 (28.1)	*0.938*
*II*	8 (36.4)	53 (27.0)	*0.359*
*III*	4 (18.2)	62 (31.6)	*0.201*
*IV*	1 (4.5)	15 (7.7)	*0.601*
**Intraoperative mortality, n (%)**	0 (0)	0 (0)	
**Anastomotic leakage, n (%)**	2 (9.1)	19 (9.7)	*0.928*
**Postoperative mortality, n (%)**	6 (27.3)	17 (8.7)	*0.011*
**Anastomotic stricture, n (%)**	2 (9.1)	33 (16.8)	*0.357*

*Absence of histologically identifiable residual cancer.

^+^Pathological stage after surgery.

### 3.3 Chemoradiotherapy

A total of 109 patients received CRT or RT alone: 19 in the cirrhotic group and 90 in the non-cirrhotic group. The cirrhotic group was younger at the time of diagnosis (median, 64 [range, 46–87] vs. 71 [range, 41–89] years; *p* = 0.022). No significant differences were found in cancer location, stage, or chemotherapy regimen between these groups. The proportions of patients who underwent RT alone and the proportions of patients who did not complete the CRT schedule due to side effects were similar between the two groups. Achievement of cCR after CRT was higher in the cirrhosis group (84.2 % vs. 43.3 %, *p* = 0.004), especially in patients with stage III disease (90.0 % vs. 35.9 %, *p* = 0.043). Among patients who achieved cCR, four patients (2 patients in each group) experienced cancer recurrence during follow-up, resulting in three eventual deaths and one receiving supportive care. The incidence of radiation-induced strictures was also higher in the cirrhotic group (26.3 % vs. 7.8 %; *p* = 0.027) (Table 3).

**Table 3:** Comparison of two groups that underwent chemoradiotherapy.

	**Cirrhosis** **(n = 19)**	**Non-Cirrhosis** **(n = 90)**	***p* value**
**Age, median (range) years**	64 (46 ∼ 87)	71 (41 ∼ 89)	*0.083*
**Male, n (%)**	19 (100.0)	79 (87.8)	*0.238*
**Child**–**Pugh Classification, n (%)**			
*A*	15 (78.9)	non-applicable	
*B*	2 (10.5)	non-applicable	
*C*	2 (10.5)	non-applicable	
**Location, n (%)**			
*Cervical*	4 (21.1)	15 (16.7)	*0.648*
*Upper thoracic*	2 (10.5)	18 (20.0)	*0.342*
*Mid thoracic*	5 (31.2)	32 (35.6)	*0.442*
*Lower thoracic*	8 (42.1)	25 (27.8)	*0.221*
cStage*, n (%)			
*I*	3 (15.8)	6 (6.7)	*0.203*
*II*	4 (21.1)	21 (23.3)	*0.830*
*III*	10 (52.6)	39 (43.3)	*0.461*
*IV*	2 (10.5)	24 (26.7)	*0.150*
**Concurrent chemoradiotherapy, n (%)**	16 (84.2)	73 (81.1)	*0.752*
***Chemotherapy regimens***			
*5-Fluouracil + Cisplatin*	11 (68.8)	54 (74.0)	*0.752*
*Paclitaxel + Carboplatin*	5 (31.2)	9 (12.3)	*0.070*
*Others*	0 (0)	10 (13.7)	*0.250*
**Radiotherapy alone, n (%)**	3 (15.8)	17 (18.9)	*0.752*
**Schedule completed, n (%)**	18 (94.7)	81 (90.0)	*0.523*
**Death during treatment, n (%)**	0 (0)	2 (2.2)	*0.951*
**Clinically complete response, n (%)**	16/19 (84.2)	39/90 (43.3)	*0.004*
According to cStage*			
*I*	2/3 (66.6)	6/6 (100.0)	*0.252*
*II*	4/4 (100.0)	17/21 (81.0)	*0.596*
*III*	9/10 (90.0)	14/39 (35.9)	*0.043*
*IV*	1/2 (50.0)	2/24 (8.3)	*0.133*
**Post-radiation stenosis, n (%)**	5 (26.3)	7 (7.8)	*0.027*

*Clinical tumor–node–metastasis (cTNM) according to American Joint Committee on Cancer (AJCC) TNM version 8.

### 3.4 Endoscopic resection

A total of 57 patients underwent endoscopic resection: 8 in the cirrhotic group and 49 in the non-cirrhotic group. Only three patients in the non-cirrhotic group underwent endoscopic mucosal resection, and all remaining patients underwent ESD. Despite longer procedural durations in the cirrhotic group (median, 35 vs. 22 min, *p* = 0.510), the difference was not statistically significant. Hospitalization durations (median, 4 days vs. 4 days; *p* = 0.146) did not differ between the two groups. Postprocedural complications (i.e., one hemorrhage and one perforation) occurred only in the non-cirrhotic group. No deaths were associated with endoscopic resection. The complete resection (75.0 % vs. 76.1 %, *p* = 0.947) and curative resection (62.5 % vs. 60.9 %, *p* = 0.930) rates did not differ between the groups. Among the three patients with cirrhosis who underwent non-curative resection, only one underwent additional RT. Of the 18 non-cirrhotic patients who underwent non-curative resection, three patients underwent additional esophagectomy and six patients underwent RT.

### 3.5 Survival

The median follow-up was 12.5 (range, 1.2–147.6) months for the cirrhotic group and 18.2 (range, 0.6–182.2) months for the non-cirrhotic group. Among surviving patients (30 cirrhotic and 209 non-cirrhotic), the median follow-up was 46.6 (range, 8.5–147.6) months for cirrhotic patients and 55.3 (range, 5.5–182.2) months for non-cirrhotic patients.

**Fig. 1:**
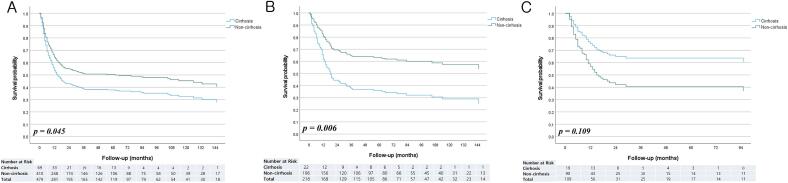
Overall survival curves between the two groups. **A)** Survival between the two groups for all patients. **B)** Survival between the two groups after surgery. **C)** Survival between the two groups after chemoradiotherapy.

Overall patient survival was lower in the cirrhotic group (HR = 1.41 [95 % CI 1.01–1.99], *p* = 0.045) (Fig. 1A). Postoperative survival was significantly lower in the cirrhotic group (HR = 2.23 [95 % CI 1.26–3.9, *p* = 0.006) (Fig. 1B). Conversely, survival after CRT was longer in the cirrhotic group but not statistically different (HR = 0.50 [95 % CI 0.21–1.17, *p* = 0.109) (Fig. 1C). The overall survival rates at 1, 3, and 5 years postoperatively were 58.7 %, 44.1 %, and 44.1 %, respectively, in the cirrhotic group and 81.0 %, 63.8 %, and 62.4 %, respectively, in the non-cirrhotic group. Overall survival rates after CRT at 1, 3, and 5 years were 73.7 %, 68.0 %, and 68.0 %, respectively, in the cirrhotic group, and 57.0 %, 39.8 %, and 39.8 %, respectively, in the non-cirrhotic group.

### 3.6 Outcomes and survivals of cirrhotic patients

**Fig. 2:**
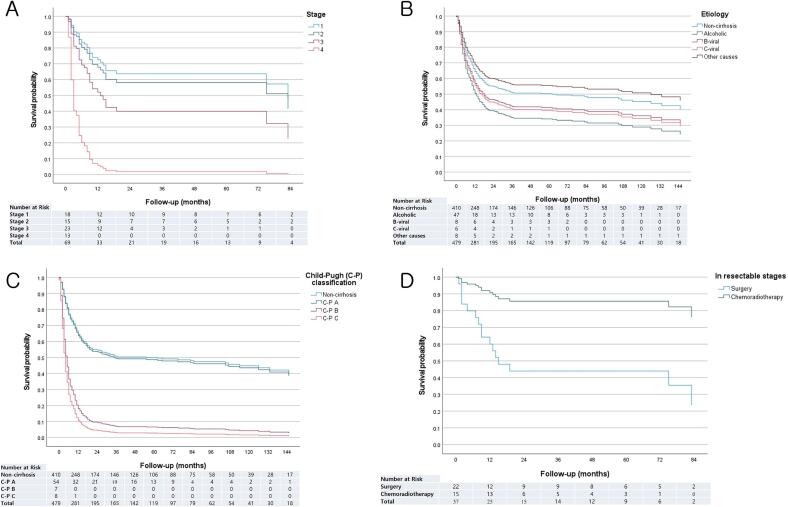
Overall survival curves for the cirrhotic group. **A)** Survival according to stages. **B)** Survival according to etiology of cirrhosis. **C)** Survival according to severity of cirrhosis. **D)** Survival according to treatment in resectable stages. (C−P, Child−Pugh).

**Fig. 3:**
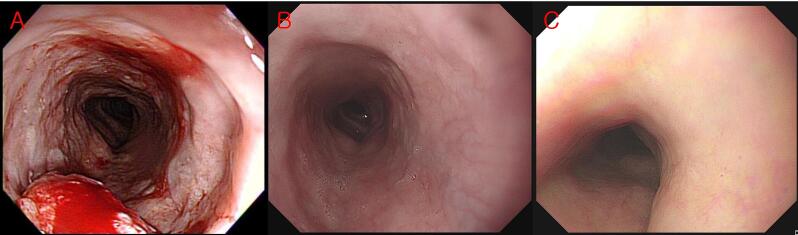
A case of a patient with cirrhosis who underwent concurrent chemoradiotherapy for esophageal squamous cell carcinoma. **A)** Esophageal squamous cell carcinoma located at the upper thoracic esophagus was observed in the endoscopic image of a 64-year-old male patient with alcoholic cirrhosis and esophageal varices. **B)** The patient received concurrent chemoradiotherapy with cisplatin and 5-fluouracil, and only a scar was observed in the similar location in the endoscopy after one year. **C)** Even after 4 years, it is still observed as a complete remission state.

The survival of 69 patients with cirrhosis according to stage and etiology of cirrhosis is illustrated in Fig. 2A and 2B. Alcoholic cirrhosis had the worst prognosis, with significantly lower survival than non-cirrhotic patients (HR = 1.57 [95 % CI 1.05–2.33], *p* = 0.027). Upon comparing survival according to C–P classification (Fig. 2C), C–P class A patients had similar survival to non-cirrhotic patients (HR = 1.04 [95 % CI 0.69–1.56], *p* = 0.864), while class B and class C patients had significantly lower survival rates (*p* = 0.001 and *p* < 0.001). Analysis of patients with cirrhosis with only resectable stage disease, excluding stage 4 patients and those who received only RT (Table 4), revealed that CRT yielded better overall survival compared to surgery (HR = 0.19 [95 % CI 0.42–0.84], *p* = 0.029) (Fig. 2D). A case of a patient having ESCC with cirrhosis who received CRT and achieved complete remission is shown in Fig. 3.

**Table 4:** Comparison between chemoradiotherapy and surgery in resectable stages in patients with cirrhosis.

	**Chemoradiotherapy (n=15)**	**Surgery (n=22)**	***p* value**
**Age, median (range) years**	71 (57 ∼ 87)	61 (51 ∼ 79)	*0.050*
**Male, n (%)**	15 (100)	20 (90.1)	*0.402*
**Child−Pugh Classification, n (%)**			
*A*	12 (80.0)	20 (90.1)	*0.351*
*B*	1 (6.7)	2 (9.1)	*0.792*
*C*	2 (13.3)	0 (0)	*0.182*
**Presence of esophageal varices**			
**Location, n (%)**			
*Cervical*	2 (13.3)	2 (1.0)	*0.685*
*Upper*	1 (6.7)	1 (4.5)	*0.781*
*Middle*	5 (33.3)	17 (77.3)	*0.010*
*Lower*	7 (46.7)	4 (18.2)	*0.070*
cStage*, n (%)			
*I*	2 (13.3)	6 (27.2)	*0.321*
*II*	3 (20.0)	9 (40.9)	*0.190*
*III*	10 (66.7)	7 (31.8)	*0.042*
**Treatment related death, n (%)**	0 (0)	6 (27.3)	*0.097*
**Remnant or recurrence, n (%)**	2 (13.3)	4 (18.2)	*0.696*
**Post-treatment stricture, n (%)**	4 (26.7)	2 (9.1)	*0.171*

*Clinical tumor−node−metastasis (cTNM) according to American Joint Committee on Cancer (AJCC) TNM version 8.

## 4 Discussion

This study revealed that when ESCC occurred among patients with cirrhosis, the prognosis was worse, as expected. However, no difference was found in overall survival between C–P class A patients and those in the non-cirrhotic group. Esophagectomy in patients with cirrhosis resulted in a high mortality rate, which is consistent with other studies [7], [8]; however, when CRT or RT was administered, complete response rate and survival were better than those in the non-cirrhotic group. Analysis restricted to the resectable stages revealed that CRT elicited better overall survival compared to surgery in patients with cirrhosis. While there may have been a selection bias with more untreated patients with cirrhosis due to their poor condition, this suggests that well-selected patients with cirrhosis undergoing CRT may result in prolonged survival compared to those without cirrhosis.

The incidence of esophageal cancer is higher in Asia than in Western countries, and more than 90 % of esophageal cancers in Asia are SCC [1]. Alcohol consumption is a major cause of ESCC and is often linked with cirrhosis. The incidence of cirrhosis among patients with esophageal cancer is approximately 3–14 % [6], [11], [14]. In our population of SCC, 14.4 % had cirrhosis, and this rate is consistent with the literature. Despite limited studies on esophageal cancer accompanied by cirrhosis, there have been some studies on surgery, including two *meta*-analyses, and postoperative mortality rate has been reported as high as 10–30 % [7], [8], [15]. Similarly, our study found significantly higher surgical mortality in the cirrhotic group (27.3 % vs. 8.7 %, *p* = 0.011). This result was obtained even though most patients with cirrhosis who underwent surgery were classified as C–P class A (90.1 %), more often in the mid-thoracic esophageal location (77.3 %), and all patients had an esophagogastric anastomosis.

Typically, surgery is the primary treatment for solid tumors; however, for SCC, RT can be curative due to its radiation sensitivity. For ESCC, evidence suggests that active surveillance without surgery is viable if a cCR is achieved after CRT [3], [16], [17]. In our study, 109 patients (19 with and 90 without cirrhosis) were followed receiving CRT, and 55 (16 with and 39 without cirrhosis) achieved cCR. Among patients who underwent surgery after CRT, 3 of 5 patients in the cirrhotic group and 11 of 19 patients in the non-cirrhotic group achieved pCR. The reason behind the higher proportion of patients with cirrhosis achieving complete response to CRT is unclear. Better survival may be due to selection bias, since only patients in good condition among the patients with cirrhosis were treated; however, this does not explain complete response. Similarly, esophageal strictures after CRT occurred more frequently in the cirrhosis group (26.3 % vs. 7.8 %, *p* = 0.027).

In our study, patients with cirrhosis were diagnosed with esophageal cancer at a younger age, and it is thought to be due to earlier initiation of tests such as endoscopy. However, no difference was revealed in tumor stage between the two groups. For surgical patients, no difference was found in age compared to the non-cirrhotic group, with most patients under C–P class A; however, the surgical mortality rate was very high in the cirrhotic group. Patients who received CRT were younger in the cirrhosis group, which may be attributed to patient selection that led to a good survival rate. For endoscopic resection, no complications or deaths were found in the cirrhotic group due to strict patient selection.

This study has some limitations. This was a single-center retrospective study, and patients with both cirrhosis and esophageal cancer were in poor condition; hence, biases in treatment selection were prominent. Second, although both cirrhosis and esophageal cancer share alcohol as a contributing cause, it is rare for both diseases to coexist; hence, the number of patients in the cirrhotic group was small. Third, the reasons behind higher rates of cCR and esophageal strictures after CRT in the cirrhosis group were not determined. Fourth, because this study entailed analysis of retrospective data collected over a relatively long period of time, the criteria for treatment selection were not unified.

## 5 Conclusion

ESCC patients with cirrhosis have a worse prognosis compared to ESCC patients without cirrhosis. However, C–P class A cirrhotic patients with ESCC have a similar prognosis to non-cirrhotic patients with ESCC. Surgery poses significant risks in the management of ESCC among patients with cirrhosis, whereas the outcomes and survival were improved with CRT or RT. Therefore, CRT may represent a preferable treatment option for appropriately selected patients with ESCC and cirrhosis.

## Funding

None.

## CRediT authorship contribution statement

**Dae Gon Ryu:** Formal analysis, Investigation, Methodology, Writing – original draft. **Mi Sook Yun:** Data curation, Methodology. **Hongqun Liu:** Investigation, Validation. **Samuel S. Lee:** Supervision, Project administration, Validation. **Sangjune Laurence Lee:** Supervision, Writing – review & editing.

## Declaration of Competing Interest

The authors declare that they have no known competing financial interests or personal relationships that could have appeared to influence the work reported in this paper.
